# Solitary fibrous tumor of the breast: A case report

**DOI:** 10.1016/j.ijscr.2023.108369

**Published:** 2023-06-01

**Authors:** Chikako Hirose, Tetsu Hayashida, Junichi Saito, Akiharu Kubo, Shuji Mikami, Hiromitsu Jinno

**Affiliations:** a Department of Surgery, Inagi Municipal Hospital, Tokyo, Japan; b Department of Surgery, Keio University School of Medicine, Tokyo, Japan; c Department of Dermatology, Keio University School of Medicine, Tokyo, Japan; d Department of Diagnostic Pathology, Keio University Hospital, Tokyo, Japan; e Department of Surgery, Teikyo University School of Medicine, Tokyo, Japan

**Keywords:** Breast, Solitary fibrous tumor, Dermatofibrosarcoma protuberans, STAT6, COL1A1-PDGFB fusion gene, Soft tissue tumor

## Abstract

**Introduction:**

Solitary fibrous tumors (SFTs) are uncommon mesenchymal neoplasms that comprise <2 % of all soft tissue tumors. They are a diagnostically challenging group of neoplasms that can occur essentially anywhere. Molecular or genetic testing of soft tissue tumors will increasingly add to the foundation of distinctive histologic features, as accurate diagnosis is critical for appropriate treatment.

**Case presentation:**

A 28-year-old woman was referred to our hospital for a left breast mass. Ultrasonography showed an oval hypoechoic mass with partially obscured boundaries. Surgical specimens revealed spindle tumor cells surrounding the mammary ducts and were immunoreactive for both CD34 and STAT6, suggesting SFTs. However, the infiltration of spindle tumor cells into the surrounding fat, and the storiform-like pattern made us consider dermatofibrosarcoma protuberans (DFSP) as a differential diagnosis. Lack of amplification of the COL1A1-PDGFB fusion gene, a characteristic feature of DFSP, led to our definitive diagnosis of breast SFT.

**Discussion:**

The presence of STAT6 in tumor cell nuclei is a highly sensitive immunohistochemical marker for SFT. In our case, morphological features evoked the differential diagnosis of DFSP and we investigated the COL1A1-PDGFB fusion gene. The diagnostic process of reliably performing careful morphological examination and immunohistochemical marker test, and then obtain conviction by molecular cytogenetic technique is more and more important for soft tissue tumors.

**Conclusions:**

We report a quite uncommon case of breast SFT and excluded DFSP as a differential diagnosis. If it is difficult to distinguish between these diseases, molecular cytogenetic analysis would be required for accurate diagnosis.

## Highlights

• Solitary fibrous tumor arising at the breast is quite uncommon
• Both solitary fibrous tumor and dermatofibrosarcoma protuberans are classified as fibroblastic/myofibroblastic soft tissue tumors
• Solitary fibrous tumor is highly immunoreactive for STAT6
• COL1A1-PDGFB fusion gene is a characteristic feature of dermatofibrosarcoma protuberans
• Molecular cytogenetic technique would be required and be helpful for accurate diagnosis of soft tissue tumors

## 1 Introduction

Solitary fibrous tumors (SFTs) are uncommon mesenchymal neoplasm that often occurs in middle-aged adults and affect both sexes equally [1]. SFT was explicitly described in 1931 as a pleural-specific tumor [2]. However SFTs are now known as ubiquitous fibroblastic tumors. Extrapleural SFTs may be found any location; 40 % are found in subcutaneous tissue, while others arise in the deep soft tissue of the extremities or in the head and neck region, thoracic wall, mediastinum, pericardium, abdominal cavity and retroperitoneum. Other affected sites include meninges, thyroid, lung, liver, gastrointestinal tract, kidney, urinary bladder, prostate, and bone [1]. There are no pathognomonic imaging findings to date, which contributes to the fact that these tumors remain difficult to diagnose and are not recognized.

Most SFTs were previously termed hemangiopericytomas (HPCs) that proposed by Stout and Murray in 1942 as “vascular tumor arising from Zimmerman's pericytes” [3], because SFTs show prominent hemangiopericytoma-like branched vascular pattern as well as HPCs. However HPC was gradually considered outmoded synonym for SFT [4]. The 4th edition of World Health Organization (WHO) classification of extrapleural solitary fibrous tumor obsoleted HPC [1].

Conventional diagnosis of SFT was based on distinctive morphological features. Typical SFT shows spindle cell proliferation exhibiting a pattern-less architecture and hemangiopericytoma-like vasculature. Tumor cells are highly immunoreactive for CD34, vimentin, bcl-2, and CD99 [5], but these antigens are not specific. Molecular analysis has revealed that the NAB2-STAT6 fusion gene is responsible for SFT [6,7]. Therefore, detection of STAT6-positive tumor cell nuclei would be useful for diagnosis of SFT [8,9]. Most SFTs are benign and can be cured by surgical resection, with a small subset exhibiting malignant feature. Malignant SFTs are usually hypercellular lesions, showing at least focally moderate to marked cytological atypia, necrosis, numerous mitoses, and infiltrative margins [1].

On the other hand, dermatofibrosarcoma protuberans (DFSP) is conventionally known to be a malignant soft tissue tumor that grows over the entire body. DFSP was originally described in conjunction with skin tumors in the WHO classification, but from the 4th edition it is included in soft tissue tumors and classified as an intermediate tumor of fibroblastic/myofibroblastic differentiation [4]. When the morphologic features of the SFT overlap with DFSP, the differential diagnosis cannot be definitively denied from histological findings alone. This report is in line with the SCARE criteria [10].

## 2 Case presentation

A 28-year-old woman visited our hospital due to a palpable painless lump in her left breast. Eight years ago she had visited another hospital for the same reason. At that time, the nodule was about 1 cm in size, and cytological examination suggested a benign tumor. Recently, during her lactation period for her second child, she visited our hospital because the size of the lump had increased. She had no family history of breast or ovarian cancer and no medical history worth considering. Physical examination revealed a relatively well-circumscribed mass measuring about 2 × 1 cm in the upper outer quadrant of her left breast, at a distance of 7 cm from the nipple. There were no other symptoms such as skin alterations, nipple discharge, or lymph node swelling in the axillary area.

**Fig. 1:**
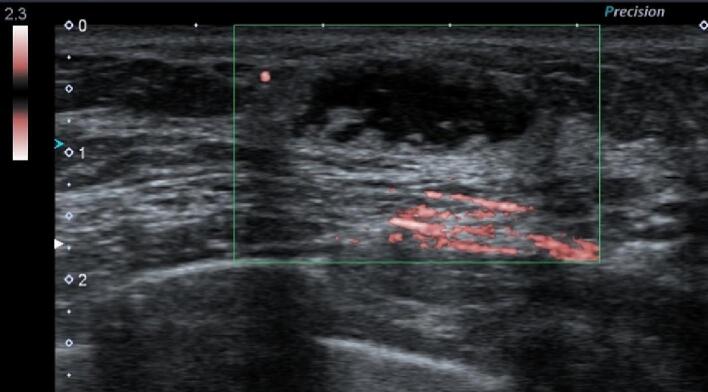
The breast ultrasound shows an oval hypoechoic mass accompanied by the front and lateral boundaries that are partially obscured. Internal heterogeneity and posterior echo enhancement are also revealed.

**Fig. 2:**
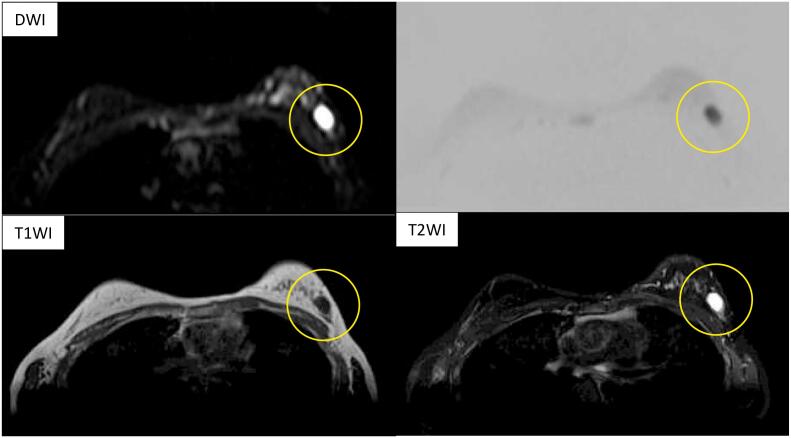
Magnetic resonance imaging reveals a relatively clear tumorous lesion with the high-intensity signal on T2WI and DWI, and the low-intensity signal on T1WI.

Bilateral mammography showed no significant findings. Breast ultrasonography showed an oval hypoechoic mass with partially obscured anterior and lateral borders. This mass had internal heterogeneity, posterior echo enhancement, and poor blood flow (Fig. 1). Contrast MRI (magnetic resonance imaging) showed a low-intensity signal on the T1-weighted image, a uniform high-intensity signal on the T2-weighted image, and an abnormal high-intensity signal on the diffusion-weighted image (Fig. 2). The mass was classified as category 3 upon cross-referencing with the Breast Imaging and Reporting Data System.

Fine needle aspiration cytology revealed small clumps of degenerated ductal cells, and aggregate of adipocytes and stromal cells. There was no evidence of malignancy. The core needle biopsy showed proliferation of spindle cells with mild atypia. Spindle cells were arranged without any specific pattern in a fibrous stroma. In hypercellular areas, tumor cells proliferated in a bundled arrangement, and some had infiltrated the surrounding fat in a storiform-like pattern. Immunohistochemically, the tumor cells were positive for CD34 and STAT6. We suspected SFT and planned excisional biopsy. As we were concerned about the differential diagnosis of DFSP morphologically, the tumor was excised with a margin of 1 cm along with the surrounding tissue, and the skin directly above the tumor and the pectoralis major fascia were sufficiently excised. No oncoplastic approach as volume replacement was required, because the tumor was located in the supra-marginal border of the mammary gland and volume of the specimen (4.3 × 3.6 cm) was small.

**Fig. 3:**
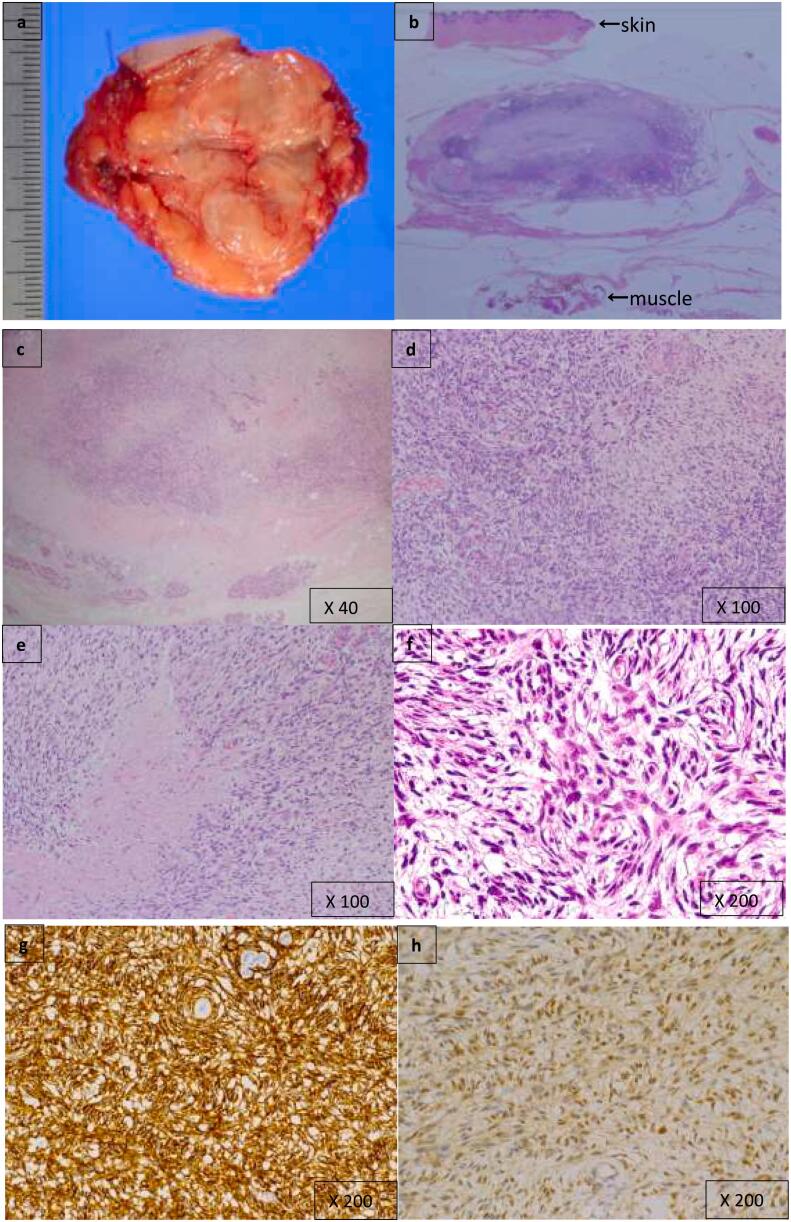
Gross appearance of cutting section of the surgical specimen (a). H&E stained section of the cutting surgical specimen (b). Tumor cells invade the mammary gland and proliferate unclearly at the boundary (c). A region with high cellularity (d). Collagen fibers are predominant in the low cellularity area (e). Striform-like pattern (f). Spindle cells are immunoreactive for CD34 (g) and STAT6 (h).

Macroscopic inspection of the surgical specimen revealed a well-circumscribed, partially encapsulated mass. Tan lesions with scattered gray-white consolidations were observed (Fig. 3a, b). Histological examination of the surgical specimen showed that spindle cells with mild atypia had proliferated invasively, and arranged into irregular bundles. Residual breast ducts and lobules were entrapped within the lesion (Fig. 3c). The tumor had a relatively monomorphic aspect with alternating hypercellular (Fig. 3d) and hypocellular areas. In the hypocellular areas, collagenized stroma was predominant (Fig. 3e). Mitosis was rare, and nuclear pleomorphism, necrosis, vascular invasion, and hemorrhages were absent in both areas. Immunohistochemically, the tumor cells were positive for CD34 (Fig. 3g) and STAT6 (Fig. 3h), and negative for smooth muscle actin, desmin, S100-protein, and epithelial membrane antigens. The overall tumor MIB-1 index was <1 %.

**Fig. 4:**
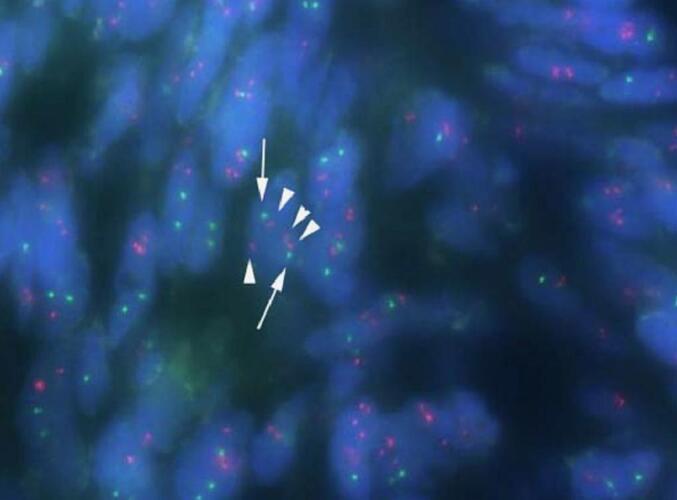
Amplification of the COL1A1-PDGFB fusion gene is not observed by fluorescence in situ hybridization (arrowhead; red: COL1A1 gene, arrow; green: PDGFB gene). (For interpretation of the references to colour in this figure legend, the reader is referred to the web version of this article.)

Based on the morphological and immunohistochemical features of the tumor, SFT was suspected. However, as a storiform-like pattern (Fig. 3f) was observed in the hypercellular areas, leading to a differential diagnosis of DFSP. The presence of the COL1A1-PDGFB fusion gene, a characteristic feature of DFSP, was evaluated by fluorescence in situ hybridization. We confirmed the absence of the COL1A1-PDGFB fusion (Fig. 4) and DFSP was denied. There has been no evidence of recurrence after a follow-up period of 60 months.

## 3 Discussion

Case reports of breast SFT are rare and have been reported in 27 English literatures to date in our knowledge (summarized in Table 1 [[11], [12], [13], [14], [15], [16], [17], [18], [19], [20], [21], [22], [23], [24], [25], [26], [27], [28], [29], [30], [31], [32], [33], [34], [35], [36], [37]]).

**Table 1:** Summary of breast SFTs reported in English literature.

Year	Author	Reference no.	Age/sex	Microscopic features in H&E stained section	Immunohistochemistry														
					STAT6	CD34	Ki67	Bcl2	CD99	Vimentin	SMA	Desmin	Actin	S100	Cytokeratins	βcatenin	p63	ER	Others
2023	Our case		28/F	Striform pattern	+	+	<1 %	+		+	−	−		−	−				
2022	Kawaguchi S	[11]	73/M	Arrangement without pattern	+	+	low												
2022	Ben Ghashir NS	[12]	54/M	Patternless architecture	+	+				+	−	−		−	−				
2021	Nitta T	[13]	78/F	Hemangiopericytoma-like growth pattern	+	+	50 %	+											
2020	Dubois C	[14]	63/F	Patternless or storiform pattern	+	+	3 %				−	−	−	−	−	−	−		
2019	Barco I	[15]	72/F	Stag horn pattern	+	+	20 %	+	+	+		−		−	−	−	−	−	Myosin-
2019	Jung MJ	[16]	75/F	Staghorn-like branching pattern	+	+		+						−	−		−		
2018	Salemis NS	[17]	42/F	Staghorn-like vessels		+	<1 %			+			−	−	−			−	
2018	Song HS	[18]				+								−				−	
2018	BrenesJ	[19]	38/F	Patternless pattern architecture		+	30 %	+	+		+	−		−	−			−	
2018	Magro G	[20]			+	+				+	−	−			−	−	−		
2018	Magro G	[21]	58/F		+	+				+	−	−							
2016	Magro G	[22]	62/F	Haphazardly arranged within a fibrous stroma	+	+		+	+	+	−	−		−				+	
2002	Magro G	[23]	81/M	Haphazard to storiform arrangement		+		Hetero +	Hetero +	+	−	−						−	
			68/M			+		+	Hetero +	+	Hetero +	Focal +						+	
2018	Park BN	[24]	63/F	Haphazardly arranged within a fibrous stroma				+			−	−		−					
2017	Tsai SY	[25]	41/F	Hemangiopericytic vascular framework															
2017	Riola-Parada C	[26]	38/F			+	30 %	+	+				+						
2016	Rhee SJ	[27]	53/M	Hemangiopericytoma-like area		+					−		−	−					
2015	Han Y	[28]	50/F		+	+		+	+										
2014	Yang LH	[29]	52/F	Staghorn vasculature, hemangiopericytoma growth pattern		+	15 %	+	+	+	−	−	−	−	−	−	−		
2010	Wignall OJ	[30]																	
2008	Rovera F	[31]	49/M	Bland looking cells admixed with thin collagen fibers		+					−	−		−	−				
2008	Meguerditchian AN	[32]	79/M			+		+		+	Focal +	Focal +	Focal +	−	−				
2004	Falconieri G	[33]	58/F	Hemangiopericytiod vascular framework, haphazardly arrangement		+		−			−								
			62/F			+		+			−								
			64/F			+		−			Focal +								
2003	Bombonati A	[34]	88/F			+						−	−	−	−			+	
2001	Salomao DR	[35]	Mean age 75/all female			+						−	−	−	−				CD31-
						+						−	−	−	−				
						+						−	−	−	−				
						+						−	−	−	−				
1997	Khalifa MA	[36]	53/F	Hemangiopericytoma-like pattern, patternless pattern		+				+	−		−	−	−				
1994	Damiani S	[37]	63/M			+				+	−	+							
			68/M			−					−	−							
			45/M			+				+	+	+							

Accurate preoperative diagnosis of breast SFT is difficult because there are no specific radiological or cytological features associated with SFT. Mammography for breast SFT is nonspecific and may be detected as a well-defined dense mass. Ultrasound often shows well-circumscribed heterogeneous masses that appear occasionally [25]. Extrapleural SFTs usually show high-intensity signal on T2-weighted MRI images and have adequate enhancement margin [30]. There are no pathognomonic imaging findings to date, contributing to the fact that breast SFTs remain difficult to diagnose and are not recognized. It is important to discriminate it from other hyperdense lesions such as fibroadenoma, phyllodes tumor and malignant tumors. In our case, the core needle biopsy showed no epithelial component hyperplasia, especially no foliate structure, so phyllodes tumors was excluded. Neither morphological nor immunohistological findings indicated leiomyoma, and there was no necrosis, mitotic pattern, or atypicality that would suggest high-grade sarcoma. Immunohistological findings also excluded synovial sarcoma. SFT of the breast may be difficult to distinguish from other mesenchymal proliferative entities such as nodular PASH [38,39].

Macroscopically, SFT is a smooth surface tumor that often has a thin fibrous capsule and usually shows a gray-white to white tone. Histological findings of typical SFT contain alternating hypercellular and hypocellular areas, and are composed of spindle and ovoid tumor cells that have scant cytoplasm and indistinct cell borders. Moreover, typical SFTs are arranged in a pattern-less architecture. Prominent branching, hemangiopericytoma-like vasculature (staghorn vasculature), and collagenous and hyalinized stroma are characteristic features of SFTs. Mitoses are generally scarce, rarely exceeding three per 10 high-power fields. Further, bleeding or necrosis is also rare [1].

Diagnosis of SFT was previously based on its characteristic morphological features. SFT has been shown to demonstrate immunoreactivity for CD34, which is usually associated with CD99 and bcl-2 expression [28]. CD34 is a sensitive but not specific marker for SFT. In addition, several mesenchymal tumors have been reported to express both CD99 and bcl-2. Diagnostic challenges more often surface when histological findings are accompanied by unusual features. We should be aware that there has been a major change in the concept of the disease due to the fact that it is a soft tissue tumor, which led to a major revision of the guidelines. Furthermore, it is conceivable that SFT might have been treated and reported as a different disease in an age when it was not possible to reliably exclude differential diseases, due to inadequate diagnostic methods. In our case, morphologic features evoked the differential diagnosis of DFSP, investigated COL1A1-PDGFB fusion gene and ruled out DFSP. COL1A1-PDGFB fusion gene assay is not versatile and it is not necessarily required for the diagnosis of SFT.

Recent developments in molecular biological analysis have identified the NAB2-STAT6 fusion gene in the vast majority of SFTs [6,7]. This fusion protein drives aberrant expression of STAT6 in the nucleus. The presence of STAT6 in tumor cell nuclei is a highly specific and sensitive immunohistochemical marker for SFT and is useful for diagnosis [8,9]. Therefore, detecting the NAB2-STAT6 fusion gene can aid in diagnostically challenging cases, though the relevant molecular assays are not available in many laboratories. The diagnostic process of reliably performing careful morphological examination and immunohistochemical marker test, and then obtain conviction by molecular cytogenetic technique is more and more important for soft tissue tumors.

Complete surgical resection with clear margins is the most important treatment for SFT, similar to other soft tissue tumors. Benign SFT shows favorable prognosis and rarely recurs or metastasizes. Although there are no studies on post-treatment monitoring focusing on breast SFT, long-term follow-up after surgical resection is recommended.

## 4 Conclusion

We reported a quite uncommon case of breast SFT. The tumor exhibited positive staining for STAT6 and CD34, suggesting SFT. We considered the differential diagnosis of DFSP and evaluated amplification of the COL1A1-PDGFB fusion gene, the absence of this amplification led to the definite diagnosis of SFT. It is conceivable that SFT might have been treated and reported as a different disease at that time when it was not possible to reliably exclude differential diseases, due to insufficient diagnostic methods. If it is difficult to distinguish between these diseases, molecular cytogenetic analysis would be required and be helpful for accurate diagnosis.

## Abbreviations

SFT: solitary fibrous tumor
DFSP: dermatofibrosarcoma protuberans
HPC: hemangiopericytoma
WHO: World Health Organization

## Consent

Written informed consent was obtained from the patient for publication of this case report and accompanying images. A copy of the written consent is available for review by the Editor-in-Chief of this journal on request.

## Ethical approval

Case reports are exempted from ethical approval in our institution, Inagi Municipal Hospital, Tokyo, Japan.

## Funding

All authors received no financial support for the preparation of this case report.

## Author contribution

Chikako Hirose and Hiromitsu Jinno conceived this case presentation and drafted the manuscript. Chikako Hirose and Junichi Saito performed the surgery, and Chikako Hirose and Hiromitsu Jinno followed up the patient. Shuji Mikami diagnosed the disease as a pathologist. Akiharu Kubo evaluated the COL1A1-PDGFB fusion gene by fluorescence in situ hybridization as a dermatologist. Tetsu Hayashida supervised this case presentation.

## Guarantor

Chikako Hirose is the guarantor of this article.

## Registration of research studies

Not applicable.

## Provenance and peer review

Not commissioned, externally peer-reviewed.

## Declaration of competing interest

All authors declare that they have no competing interests.
